# Hypothalamus volume mediates the association between adverse childhood experience and PTSD development after adulthood trauma

**DOI:** 10.1038/s41398-023-02576-2

**Published:** 2023-08-04

**Authors:** Hong Xie, Chia-Hao Shih, Sulaiman D. Aldoohan, John T. Wall, Xin Wang

**Affiliations:** 1 Department of Neurosciences, University of Toledo, Toledo, OH USA; 2 Department of Emergency Medicine, University of Toledo, Toledo, OH USA; 3 Department of Radiology, University of Toledo, Toledo, OH USA; 4 Department of Psychiatry, University of Toledo, Toledo, OH USA

**Keywords:** Psychiatric disorders, Human behaviour

## Abstract

The hypothalamus is critical for regulation of the hypothalamic-pituitary-adrenal (HPA) axis and response to stress. Adverse childhood experience (ACE) can affect brain structure, which may contribute to development of posttraumatic stress disorder (PTSD) after subsequent adult trauma. It is unclear, however, if ACE history is particularly associated with aspects of hypothalamic structure which contribute to development of PTSD. To address this issue, the present study longitudinally assessed hypothalamic volumes and their associations with ACE and early post-trauma stress symptoms in subjects who did or did not develop PTSD during 12 months after adult trauma. 109 subjects (18–60 years, F/M = 75/34) completed the PTSD Checklist (PCL) questionnaire for post-trauma stress symptoms, the Childhood Trauma Questionnaire (CTQ) for ACE assessment, and an initial MRI brain scan for hypothalamic volume measurement, within 2 weeks after adult trauma. At post-trauma 12 months, subjects underwent a subsequent PTSD diagnosis interview using the Clinician-Administered PTSD Scale (CAPS), and a follow-up MRI scan. Left and right hypothalamus volumes at 2 weeks after adult trauma negatively correlated with CTQ scores. Right hypothalamus volume at this early time mediated an association between ACE and PTSD symptoms 12 months later. Right hypothalamus volumes also remained persistently smaller from 2 weeks to 12 months after trauma in survivors who developed PTSD. These results suggest that smaller right hypothalamus volume may be related to ACE history in ways that contribute to PTSD development after trauma in adulthood.

## Introduction

About 7% of Americans suffer from posttraumatic stress disorder (PTSD) at some point in their lives [1]. PTSD is debilitating and characterized by intrusive memories of the traumatic event, avoidance of trauma-associated circumstances, negative mood and cognition, and hyperarousal symptoms that persist more than one month after trauma [1, 2]. PTSD has serious impacts on public health; however, effective PTSD treatment remains a clinical challenge [3, 4]. New insight into brain conditions that underlie PTSD development is clearly needed.

The hypothalamic-pituitary-adrenal (HPA) axis plays a critical role in stress responses to traumatic events, and impairments in HPA axis structure or function can lead to deficits in stress response and development of PTSD [5, 6]. The hypothalamus, a key component of the HPA axis, receives broad inputs from the brain, including from, e.g., hippocampus, amygdala, frontal cortex, thalamus, and brainstem [5, 7, 8]. High numbers of corticotropin-releasing hormone (CRH) neurons are found in the paraventricular nucleus (PVN) of the hypothalamus. With acute stress, CRH is rapidly released to the anterior pituitary from which adrenocorticotropin hormone (ACTH) is secreted to stimulate adrenal cortisol synthesis and release to manage stress. Hypothalamic CRH neurons are targets of cortisol negative feedback that inhibits HPA axis activation [9, 10]. Associations between cortisol levels and PTSD have been studied extensively but remain incompletely understood [11, 12]. For example, urinary or salivary cortisol levels have been reported to be lower [13, 14], higher [15], or not changed [16] in PTSD groups compared to control groups. The role of the hypothalamus in regulation of cortisol in PTSD patients remains unclear.

Adverse childhood experiences (ACEs) appear to increase risk for PTSD after subsequent trauma in adulthood [17–19]. ACEs involving, e.g., physical, emotional and sexual abuse or physical and emotional neglect are known to affect 7–60% of children [17, 20–22]. Further studies suggest that ACEs may lead to dysregulation of cortisol levels [23] that contribute to PTSD development [24, 25]. However, particular ways by which ACEs affect the HPA axis and lead to PTSD development in adulthood remain unclear.

ACEs are thought to influence changes in brain development that may be associated with impairments in emotion regulation, attention, learning, and mental health [26–32]. Structural MRI (sMRI) studies suggest that chronic PTSD patients with an ACE history have smaller volumes of prefrontal and insular cortices, hippocampus, and amygdala, thus suggesting that ACE-related abnormal development of brain structure may contribute to PTSD [30, 33–36]. Our recent work has reported that ACE history was negatively associated with thalamic volume within two weeks after adult trauma and positively associated with subsequent PTSD symptom severity [37]. An early post-trauma smaller thalamic structure raises the possibility that ACE may increase risks for PTSD development by involvement of structural effects in other diencephalic components including the hypothalamus. Animal studies suggest early life stress may cause hypothalamic cell loss, genetic changes, and malfunctions [38–40]. These findings suggest a possibility that ACE may affect development of hypothalamic structure in ways that contribute to PTSD risk after adult trauma. Human MRI studies have found that hypothalamus volumes are smaller in patients with generalized anxiety disorder (GAD) [41] and in postmortem brains of patients who had major depressive disorder (MDD) [42], however, there are no human studies of potential associations between ACE, hypothalamus structure, and PTSD.

To address this possibility, the present longitudinal study assessed hypothalamic volumes in adult trauma survivors with ACE histories to investigate potential associations between ACE and hypothalamus volume, stress, and PTSD development over the initial 2 weeks – 12 months after trauma. FreeSurfer automated segmentation of the hypothalamus has been validated [43] and increasingly applied in research on neurological and psychiatric disorders [44, 45]. We used FreeSurfer hypothalamic volume measures to test the hypothesis that hypothalamic volume may mediate an association between ACE history and PTSD development after adult trauma.

## Materials/subjects and methods

### Subject enrollment and procedures

The procedures have been previously described in detail [37] and are briefly outlined here. Adult subjects (18–60 years) who were sent to a hospital Emergency Department (ED) within 48 hours after a life-threatening traumatic event were recruited. Traumatic events involved motor vehicle collision (MVC), physical assault, sexual assault, or other trauma. Excluded from the study were subjects who: (1) were severely injured or had other contraindications for MRI scanning, (2) were diagnosed with severe psychiatric or neurological problems including history of moderate or severe traumatic brain injury, (3) were under the influence of alcohol or substances at the time of trauma, or (4) reported a low level of acute pain in MVC trauma survivors (Numeric Pain Rating Scale (NPRS) < 6) [46]. All subjects included in the study gave written informed consent. Consenting subjects completed the DSM-V PTSD Checklist (PCL) [47] to assess acute post-trauma stress severity. Only survivors with high stress symptoms (PCL score ≥ 28) were enrolled to increase possibility of PTSD occurrence at 12 months after trauma. All participants completed the Childhood Trauma Questionnaire (CTQ) to retrospectively identify ACE history, and an MRI brain scan for hypothalamic volume measures, within 2 weeks after trauma. For follow-up at 12 months, survivors completed a second MRI brain scan and were interviewed by an experienced clinical psychologist for PTSD diagnosis using the Clinician-Administered PTSD Scale (CAPS). All study procedures were approved by Institutional Review Boards.

### Psychological assessments

The PCL survey is a 20 item symptom assessment [47]. The total score range of 0 – 80 reflects low to high stress symptom severity. Subjects were instructed to rate symptoms on the PCL survey with specific regard to the trauma which brought them to the ED and subsequent enrollment in the current study.

The 28 item CTQ survey was used to quantitatively and qualitatively assess ACE throughout childhood up to 18 years of age [48]. CTQ evaluates 5 types of childhood maltreatments, including emotional, physical, and sexual abuse, and emotional and physical neglect. Total CTQ score was used to reflect overall ACE history.

PTSD was diagnosed with the CAPS interview at 12 months after trauma using DSM-V criteria: at least 1 re-experiencing, 1 avoidance, 2 negative feeling, and 2 hyperarousal symptoms [49]. Survivors with at least 1 symptom in each of these symptom clusters were diagnosed with partial PTSD [50]. Survivors with full or partial PTSD diagnoses at 12 months post-trauma were included in the PTSD group. Survivors who didn’t meet PTSD diagnosis criteria at 12 months were included in the non-PTSD group as trauma-exposed controls.

### Structural MRI acquisition and processing

Survivors were scanned using a 3 T General Electric Signa HDx MRI scanner (GE Healthcare, Chicago, IL). A high-resolution T1-weighted structural MRI (sMRI) image was obtained using a previously validated high-resolution 3D FSPGR structural MRI image protocol (TR = 7.836 ms, TE = 2.976 ms, FA = 9°, NEX = 1, field of view= 256 × 256 mm, matrix = 256 × 256, slice thickness= 1 mm, voxel dimensions= 1 × 1 × 1 mm^3^, 164 contiguous axial slices) [37].

Brain sMRI images were processed using FreeSurfer program (Verson 7.2) (https://surfer.nmr.mgh.harvard.edu). The FreeSurfer automated segmentation procedure for hypothalamus was used to measure hypothalamus volumes. This procedure is based on a convolutional neural network using deep machine learning methods [43]. These automated measures have been validated to show good consistency (high Dice coefficient of 0.83 and low boundary distance) with intra-rater manual segmentation measures and to be superior to inter-rater reliabilities of manual segmentation. This approach significantly outperformed (0.07 difference in Dice scores) a commonly used multi-atlas automated segmentation approach, and was validated (low rejection rate of 0.89%) with an independent large heterogeneous dataset of 675 scans collected from a variety of MR scanners using different sequences [43]. An increasing number of studies has applied this procedure in research on neurological and psychiatric disorders [44, 45]. Intracranial volume (ICV) was also reported by FreeSurfer. FreeSurfer image processing and segmentation was visually checked by the researcher blinded to psychological assessments and CAPS interview for PTSD diagnosis.

### Statistical analyses

Distributions of the data, within-group variation, and between-group variance were inspected. Differences on hypothalamic volumes of the PTSD and non-PTSD groups were assessed using analysis of covariance (ANCOVA) with scan days since trauma as a covariate factor. In addition, trauma type was added to test the type of trauma effect on hypothalamic volume. Comparison analyses of the PTSD vs non-PTSD groups were also done for PCL, CAPS and CTQ scores using ANCOVA. Relationships between CTQ scores, hypothalamus volumes, and PTSD symptom severity were examined using partial correlations. A simple mediation model was used to test for a potential mediation effect of hypothalamus volume at post-trauma 2 weeks on the association of pre-trauma CTQ scores with CAPS scores at post-trauma 12 months. Repeated measure ANCOVA (RM-ANCOVA) was used to test the effects of time and time × group interaction on hypothalamic volume change during the post-trauma first year, adjusting for interval days between the two scans. All above tests were adjusted for the effects of age and sex as covariates, and ICV was also included as a covariate in brain volume analyses. Group comparisons of age, days since trauma until scans, and interval between scans were done using T tests. Group comparisons of sex of subjects (male and female) and trauma type were done using Chi-Square tests. Based on our previous work showing medium effect sizes of associations between ACEs, thalamic volumes, and PTSD symptom severity [37], along with an extensive simulation study on required sample size for detecting mediating effect conducted by Fritz and MacKinnon [51], a sample size of 78 was needed for detecting the expected mediation effect using percentile bootstrap test at 0.80 power. Statistical analyses were conducted using SPSS version 28 (IBM Corp., Armonk, NY) and the ‘PROCESS’ macro for SPSS [52]. Data are reported as mean ± standard deviation, with *p* < 0.05 as the significance level.

## Results

109 acute trauma survivors of MVC, physical or sexual assault, or other acute trauma completed an initial sMRI scan, longitudinal symptom assessments, and PTSD diagnosis interview at 12 months post-trauma. Subjects (*n* = 41) who were diagnosed with PTSD met full (*n* = 31) or partial (*n* = 10) PTSD diagnosis criteria and were included in a PTSD group. The remaining subjects (*n* = 68) did not meet full or partial PTSD diagnosis and were included in a non-PTSD group (Table 1). A subsample of 76 subjects (30 PTSD; 46 non-PTSD) who had a follow-up sMRI scan at 12 months after trauma were included in longitudinal analyses. There was a 27% (11 of 41) loss of follow-up scans in the PTSD group, and a 30% (22 of 68) loss of follow-up scans in the non-PTSD group due to scan incompletion during the study period or to image quality issues.

**Table 1:** Demographics, psychological assessments, trauma types and hypothalamus volume measurements.

	PTSD	non-PTSD	diff.^a^	*p*
Subject N	41	68		
Sex (Female/Male)	30/11	45/23	0.583	0.445
Age (year)^b^	35.4 ± 11.7	32.8 ± 9.8	−1.255	0.212
Post-trauma days^b^				
1st Scan	11 ± 5	10 ± 4	−1.35	0.18
2nd Scan	403 ± 60	427 ± 110	1.131	0.261
Scan interval	393 ± 60	413 ± 109	0.933	0.354
CTQ scores^b^	63.7 ± 25.2	54.0 ± 23.7	2.695	0.104
2-week PCL score^b^	55.2 ± 13.3	48.2 ± 12.2	7.639	0.007*
12-month CAPS score^b^	32.5 ± 12.4	8.0 ± 7.2	166.744	<0.001*
Trauma Type (N)^c^				
MVC	15	40	6.079	0.108
PA	20	24		
SA	4	2		
other	2	2		
Hypothalamus volume (mm^3^)^d^				
within 2 weeks after trauma				
Left hypothalamus^b^	416.4 ± 45.0	429.5 ± 40.1	1.466	0.229
Right hypothalamus^b^	397.9 ± 43.0	418.6 ± 35.2	7.786	0.006*

^a^χ^2^ test for sex and trauma type; T test for age and post-trauma days; ANCOVA for PCL, CAPS and CTQ with adjustment for age and sex; ANCOVA for hypothalamus volume with adjustment for age, sex, ICV and scan days since trauma.

^b^means ± standard deviation.

^c^the number of subjects in each trauma type. *MVC* motor vehicle collision, *PA* physical assault, *SA* sexual assault.

^d^ANCOVA, η^2^ = 0.01 (in left) and η^2^ = 0.07 (in right).

* significant at *p* < 0.05 level.

Skewness tests indicated hypothalamic volumes at post-trauma 2 weeks and 12 months were normally distributed (Supplemental Table 1). Within-group variance of hypothalamic volume was estimated and homogeneity of variances between groups was assessed by Levene’s tests (all *p* > 0.05, Supplemental Table 1). PTSD and non-PTSD groups did not differ with respect to age, sex, days since trauma for scans, interval days between scans, or trauma type (Table 1). PCL scores at 2 weeks and CAPS scores at 12 months were significantly higher in the PTSD than non-PTSD group (Table 1). CTQ scores did not significantly differ across groups (Table 1).

### Hypothalamus volumes at 2 weeks after trauma in subjects who were or were not subsequently diagnosed with PTSD 12 months later

**Fig. 1:**
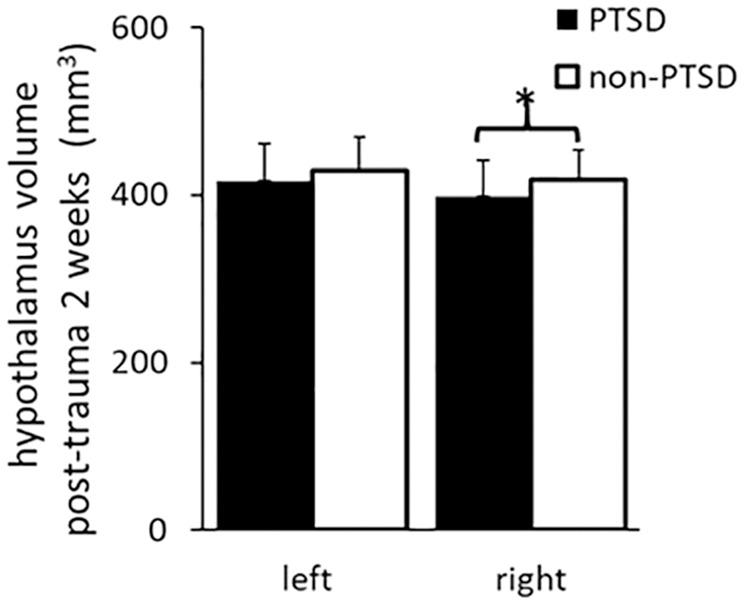
Comparison of left and right hypothalamus volumes at 2 weeks after trauma for PTSD and non-PTSD groups. Volume of right hypothalamus was significantly smaller in the PTSD (N = 41) than non-PTSD (N = 68) group. *significant level *p* < 0.05.

At 2 weeks after trauma, right hypothalamus volume was significant smaller in the PTSD group as compared to the non-PTSD group (Table 1; Fig. 1; adjusted for age, sex, ICV and days after trauma). This difference remained significant when type of trauma was also considered (F(1, 102) = 8.360, *p* = 0.005). In contrast, left hypothalamic volume was not significantly different in the PTSD vs non-PTSD group at 2 weeks after trauma (Table 1, Fig. 1).

### Relationships between preceding ACE history, post-trauma hypothalamic volumes, and PTSD symptoms

**Fig. 2:**
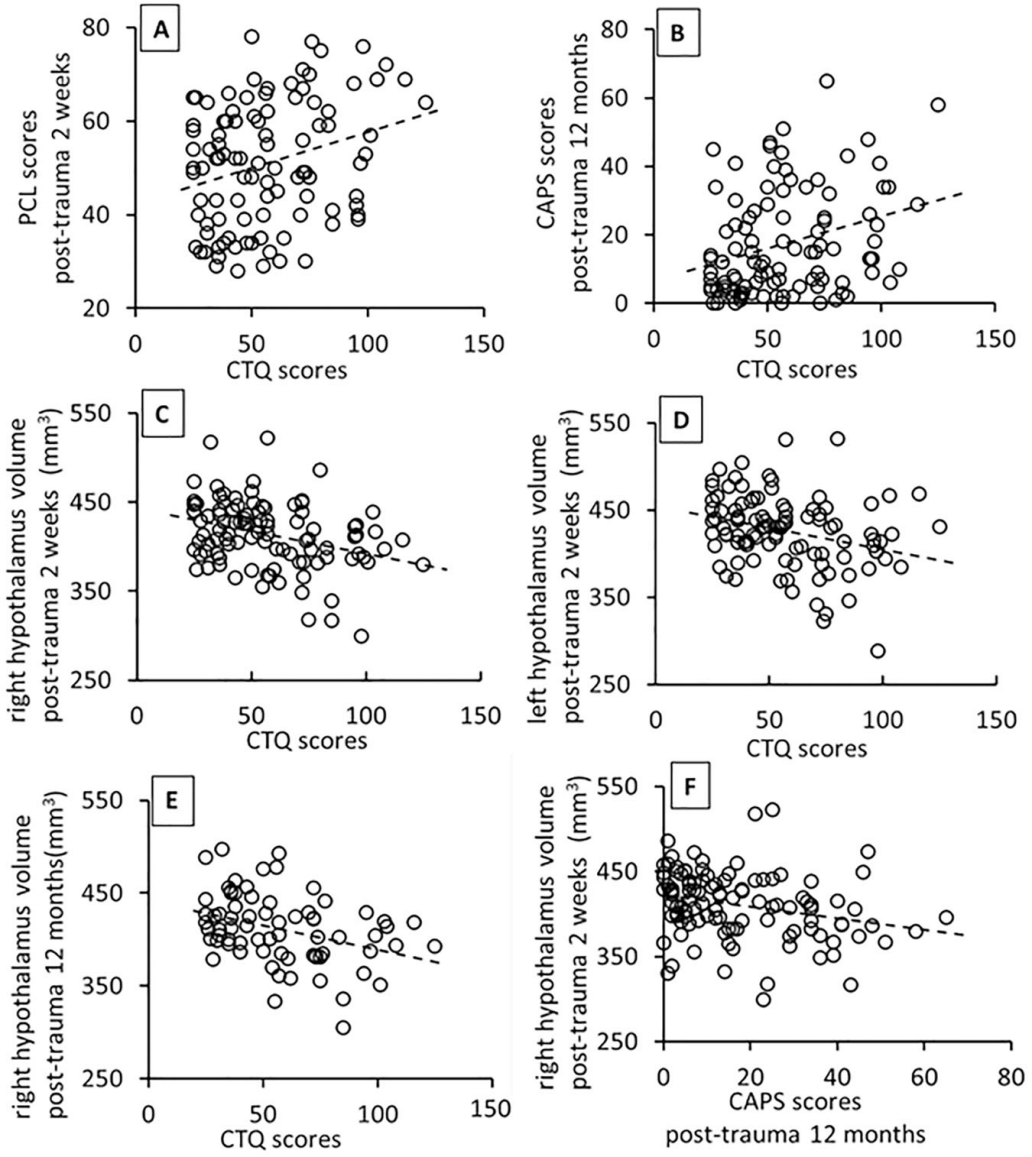
Correlations between hypothalamus volumes, PCL, CTQ and CAPS scores. CTQ scores significantly positively correlated with PCL scores at post-trauma 2 weeks **A**, and with CAPS scores at post-trauma 12-months **B**. CTQ scores significantly negatively correlated with volumes of right **C**, and left **D** hypothalamus at post-trauma 2 weeks, and with volumes of right hypothalamus **E** at post-trauma 12 months. Volumes of right hypothalamus at 2 weeks after trauma negatively correlated with CAPS scores at 12 months after trauma **F**. All correlations were adjusted for age and sex, and ICV was additionally adjusted for in hypothalamic volume analyses.

CTQ scores indicating ACE history were significantly positively correlated with PCL scores at 2 post-trauma weeks (r(96) = 0.250, *p* = 0.013, Fig. 2A), and with CAPS scores at post-trauma 12 months (r(96) = 0.274, *p* = 0.006, Fig. 2B), adjusting for age and sex. These results suggest greater ACE was related to more severe PTSD symptoms over one year after trauma. The positive correlations with post-trauma 2-week PCL and 12-month CAPS scores particularly held for trauma survivors who experienced childhood emotional, physical, or sexual abuse (Supplemental Table 2).

CTQ scores were significantly negatively correlated with both right and left hypothalamic volumes at 2 weeks after trauma (right: r(95) = −0.272, *p* = 0.007; left: r(95) = −0.240, *p* = 0.018; Fig. 2C, D). CTQ scores significantly negatively correlated with hypothalamic volumes at 12 months after trauma on the right (r(66) = −0.264, *p* = 0.030, Fig. 2E) but not left side. These results suggest more severe ACE history was associated with smaller right and left hypothalamus volumes at two weeks, and smaller right hypothalamus volume at a year, after trauma. With respect to different types of childhood trauma, childhood abuse and neglect were significantly negatively associated with post-trauma 2-week right and left hypothalamus volumes (Supplemental Table 2). At 12 months, types of childhood abuse but not neglect significantly negatively correlated with right hypothalamic volume whereas neither abuse nor neglect correlated with left hypothalamic volume (Supplemental Table 2). This suggests that different types of childhood trauma may have different effects on left and right hypothalamic volumes at different times after adult trauma.

Right hypothalamic volumes at 2 weeks after trauma significantly negatively correlated with CAPS score at 12 months after trauma (r(104)= −0.241, *p* = 0.013, Fig. 2F). This association did not hold for left hypothalamus. This suggests early post-trauma right hypothalamic volumes were associated with subsequent PTSD symptom severity.

### Hypothalamus volume at post-trauma 2 weeks mediated the association of preceding ACE with PTSD symptoms at post-trauma 12 months

**Fig. 3:**
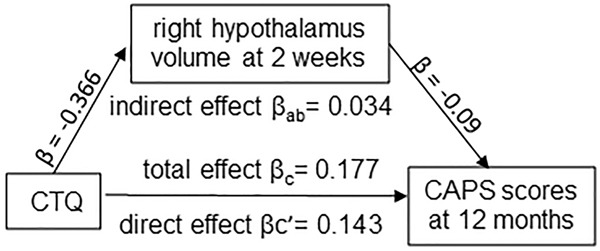
Mediation effect of right hypothalamus volumes. Analysis indicating a mediation effect of right hypothalamic volumes at post-trauma 2 weeks on an association between CTQ scores and CAPS scores at 12 months after trauma, adjusted for age, sex, and ICV.

In a simple mediation model, the total effect of CTQ score on the CAPS score was significant (β_c_ = 0.177, SE = 0.063, *p* = 0.006, 95% CI [0.051, 0.303]), and the direct effect of CTQ score on the CAPS score was significant when holding right hypothalamic volume constant (β_c’_= 0.143, SE = 0.065, *p* = 0.030, 95%CI [0.014, 0.272], Fig. 3). Additionally, the indirect effect of right hypothalamic volume on the association between CTQ score and CAPS score was significant (β_ab_ = 0.034, SE = 0.020, 95% CI [0.001, 0.080], Fig. 3). The model accounted for a significant amount of variance in PTSD symptom severity (R^2^ = 0.132, *p* = 0.019). These results suggest early post-trauma right hypothalamus volume mediated an effect of preceding ACE on PTSD symptoms 12 months after trauma.

In contrast to the above findings for right hypothalamus, the indirect effect of left hypothalamic volume at 2 weeks after trauma on the association between CTQ scores and CAPS scores at post-trauma 12 months was not significant (β_ab_ = 0.008, SE = 0.017, 95% CI [−0.024, 0.045]), although the total effect between CTQ and CAPS scores was significant (β_c_ = 0.177, SE = 0.063, *p* = 0.006, 95% CI [0.051, 0.303]) and the direct effect between CTQ and CAPS scores was significant when holding left hypothalamic volume constant (β_c’_= 0.169, SE = 0.066, *p* = 0.011, 95%CI [0.039, 0.300]). This model accounted for a significant amount of variance in PTSD symptom severity (R^2^ = 0.097, *p* = 0.045). These findings suggest that early post-trauma left hypothalamus volume did not mediate the association between ACE and PTSD symptoms at 12 months after trauma.

In summary, the mediation analyses corroborated and extended findings by providing further indications that right hypothalamic volume mediated the association between CTQ scores and subsequent CAPS scores at post-trauma one year.

### Longitudinal progression of hypothalamic volumes over 12 months after trauma in subjects who were or were not diagnosed with PTSD

**Fig. 4:**
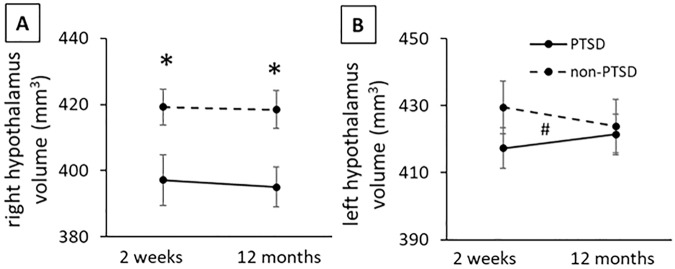
Longitudinal analysis of hypothalamus volumes at 2 weeks and 12 months after trauma in PTSD and non-PTSD groups. **A** There was a significant PTSD group effect in right hypothalamus volume, and post-hoc comparisons indicated significant smaller volumes in the PTSD vs non-PTSD group at both time points. **B** There was a significant time by PTSD group interaction in left hypothalamus volume. Repeated measure ANCOVA with age, sex, ICV and days between scans included as covariates. Group effect (*) and group x time interaction effect (^#^) were significant at *p* < 0.05 level.

For right hypothalamus, RM-ANCOVA analysis of 76 subjects with both initial and follow-up sMRI scans revealed an overall significant PTSD diagnosis group effect for right hypothalamic volume (F(1, 70) = 4.205, *p* = 0.044, µ^2^ = 0.06; Table 2; adjusted for age, sex, ICV and interval days between scans). Post hoc comparisons indicated right hypothalamic volumes were smaller in the PTSD than non-PTSD group at both 2 weeks (F(1, 70) = 4.405, *p* = 0.039, µ^2^ = 0.06) and 12 months (F(1, 70) = 4.052, *p* = 0.048, µ^2^ = 0.06) after trauma (Fig. 4A).

**Table 2:** Hypothalamus volume changes between 2 weeks and 12 months after trauma.

Hemi-sphere	post-trauma time	Volume (mm^3^)		PTSD effect^a^			Time effect^a^			Time*PTSD^a^		
		PTSD	non-PTSD	F	*p*	η^2^	F	*p*	η^2^	F	*p*	η^2^
Right	2 weeks	397.2 ± 41.9	419.2 ± 36.1	4.205	0.044*	0.06	1.046	0.310	0.02	0.019	0.889	<0.01
	12 months	395.1 ± 33.0	418.5 ± 39.1									
Left	2 weeks	417.4 ± 43.2	429.5 ± 41.5	0.153	0.697	<0.01	0.002	0.963	<0.01	5.658	0.020*	0.08
	12 months	421.4 ± 43.5	423.9 ± 40.8									

^a^RM-ANCOVA, age, sex, ICV and days between scans as covariates.

*significant at *p* < 0.05 level.

After adjusting for trauma type, group differences remained significant at 12 months (F = 4.204, *p* = 0.044) and at a trend level at 2 weeks (F = 3.908, *p* = 0.052) after trauma. Time and time × group interaction effects were not significant for right hypothalamus volume over the first post-trauma year (Fig. 4A).

For left hypothalamus, group and time effects were not significant, but the time × group interaction was significant (F(1, 70) = 5.658, *p* = 0.020, µ^2^ = 0.08; Table 2; Fig. 4B; adjusted for age, sex, ICV and interval days between scans). However, post hoc RM-ANCOVA did not show significant changes over time in left hypothalamus volume from post-trauma 2 weeks to 12 months in either the PTSD group (F = 0.155, *p* = 0.697), or non-PTSD group (F = 0.373, *p* = 0.545).

## Discussion

Potential associations between ACE and hypothalamic structure, stress, and PTSD after adult trauma have not been previously investigated. The present study provides the first longitudinal tracking of hypothalamus volume beginning early after adult trauma and its potential relationships to ACE and post-trauma PTSD development. Main findings are that at 2 weeks and 12 months post-trauma, right hypothalamic volumes, which were significantly negatively associated with both preceding ACE and post-trauma stress symptoms, were significantly smaller in trauma survivors who developed PTSD. Moreover, right hypothalamus volume at post-trauma 2 weeks mediated an association between preceding ACE and PTSD symptoms 12 months after trauma. Left hypothalamus volume at 2 weeks after trauma also negatively associated with ACE, and changes in left hypothalamus volumes over one year post-trauma differed in the survivors who did vs did not develop PTSD. These results provide evidence that the hypothalamus plays a role in development of PTSD in adult trauma survivors with an ACE history, and that right hypothalamus may be particularly important.

Taken together, the findings that right hypothalamus volume remained smaller from 2 weeks to 12 months after trauma in survivors with PTSD and that right hypothalamus volumes at 2 weeks negatively associated with PTSD symptoms at 12 months, suggest smaller right hypothalamus volume may contribute to development of PTSD over the initial year after trauma. This raises a possibility that smaller hypothalamus may be indicative of alterations in hypothalamus states. This may include, for example, loss of hypothalamic CRH neurons and related impairment of HPA axis reactions to stressful traumatic experiences. Impairment of HPA axis reactions to stress may change post-trauma emotion regulation and/or fear learning and memory consolidation that contributes to PTSD [5, 6].

Smaller right hypothalamus volume at post-trauma 2 weeks in the trauma survivors with PTSD is consistent with possibilities that volume reduction may have occurred prior to and/or soon after adult trauma. CTQ scores were negatively associated with both right and left hypothalamus volumes at post-trauma 2 weeks, which suggests ACE may have linked to hypothalamus volume reduction prior to the adult trauma. The HPA axis develops during gestation and childhood, especially in critical developmental windows during preschool and adolescence, and environmental stressors may affect development of the HPA axis over a broad age range depending on stress duration and severity [53]. This may include hypothalamus development. Stress studies suggest that long-lasting elevated cortisol due to stress may induce neuron loss, demyelination, synaptic pruning, or abnormal neurogenesis which, in turn, may be associated with HPA axis hypo-reaction to stress [38, 54–56]. For example, early life stress may have long-lasting inhibitory effects on proliferation of neural stem or precursor cells that lead to functional changes in hypothalamus [38]. Prolonged cortisol-induced glucocorticoid receptor (GR) expression and functional alterations are also associated with hypothalamic changes [54, 57]. A human magnetic resonance spectroscopy (MRS) study reported lower N-acetyl- aspartate/creatine ratios in pediatric PTSD patients. N-acetyl- aspartate (NAA) is a marker of neural integrity, and a low NAA/creatine ratio may reflect neuron loss in brain regions including hypothalamus [58]. It is feasible that long-lasting alterations in HPA axis neuron loss and functions may be part of ACE contributions to abnormal hypothalamus development. As an alternative to developmental changes prior to adult trauma, there is evidence that volumes of brain structures other than hypothalamus can change during initial weeks after trauma [59]. Further studies are needed to distinguish pre-trauma developmental vs post-trauma rapid changes in hypothalamus structure in trauma survivors with ACE history.

Right hypothalamus volume at 2 weeks after trauma mediated a positive association between ACE and PTSD severity at one year after trauma. Positive association between ACE and PTSD symptoms has been consistently reported in previous work [17]. The mediating effect of right hypothalamus on this association supports the possibility that ACE related changes in hypothalamic structure increase risk for PTSD development after adult trauma.

In contrast to the right hypothalamus, left hypothalamus volume did not differ in trauma survivors who did vs did not develop PTSD. A significant time × group interaction raised the possibility that left hypothalamus volume increased in the PTSD group and decreased in the non-PTSD group over the year after trauma; however, these different directions of changes over time were not significant when tested separately in each group. The possibility of post-trauma progressive change is arguably consistent with the finding that the negative association between ACE and left hypothalamus volumes was significant at 2 weeks but was not significant at 12 months. The difference in findings for left vs. right hypothalamus volumes suggest the two sides of the hypothalamus may play different roles in PTSD development after adult trauma. Consistent with the possibility of different roles, right vs left hypothalamic dominance has been seen in some studies. For example, right hypothalamus is dominant for reproduction and left hypothalamus for food seeking and energy homeostasis [60]. Right hypothalamus has been reported to be dominant in regulating cardiovascular responses evoked by stressors [61]. It has been proposed that the right hemisphere mediates greater physiological response to stress [62, 63], thus, raising the possibility that right hypothalamus may be more involved in stress reactions to ACE and adult trauma. Current understanding provides limited explanation for a possible difference in left hypothalamus volume alterations over the year after trauma in survivors who did vs did not develop PTSD. Speculations are possible. For example, right hypothalamus impairments may have reduced cortisol reactions in trauma survivors with PTSD, and related decreased cortisol feedback on CRH neurons may have disinhibited left hypothalamus activation related, in turn, to enlargement of the left hypothalamus to partially compensate for a smaller right hypothalamus. However, alternative speculations are also possible and require study.

This study has several limitations. (1) Only trauma survivors with high acute stress symptoms were enrolled; therefore, the generalizability of the findings needs consideration. (2) All subjects had undergone trauma. A trauma-unexposed control group would be useful to study possible effects of trauma exposure or ACEs per se. (3) The present study does not address age at which ACE occurred or ACE duration. Normal brain development varies with age; thus, ACE associations with hypothalamic volume may vary with age at which ACE occurs. (4) ACE is associated with, e.g., depression, addiction, and other mental problems that can be comorbid factors for PTSD and that should be considered in future research. (5) Stress hormone levels were not measured. Relationships between hypothalamus volume and stress hormone levels require attention.

In conclusion, the present investigation provides the first evidence that smaller right hypothalamus volumes may mediate PTSD development in adult trauma survivors with an ACE history. Reduced volume of the hypothalamus may contribute to impairments of HPA axis reactions to stress after adult trauma and early development of PTSD. The findings suggest the hypothalamus plays an interesting role in mediating adverse childhood experiences and adult trauma associations to PTSD.

### Supplementary information

Supplementary tables
