# Supplementary material for: Hypothalamus volume mediates the association between adverse childhood experience and PTSD development after adulthood trauma
Source: Transl Psychiatry. 2023 Aug 4;13:274. doi: 10.1038/s41398-023-02576-2 (PMC10403516; doi:10.1038/s41398-023-02576-2)
Supplement: Supplementary file 1 — Supplementary tables [file 41398_2023_2576_MOESM1_ESM.docx]

| **Supplemental Table 1** Hypothalamus volume measurement distributions and variances | | | | | | | | |
| --- | --- | --- | --- | --- | --- | --- | --- | --- |
| Hypothalamus volume (mm^3^) | Post-trauma 2 weeks | | | | Post-trauma 12 months | | | |
|  | Left hemisphere | | Right hemisphere | | Left hemisphere | | Right hemisphere | |
|  | PTSD | non-PTSD | PTSD | non-PTSD | PTSD | non-PTSD | PTSD | non-PTSD |
| Skewness | -0.085 | -0.289 | 0.260 | -0.277 | 0.761 | -0.534 | 0.146 | -0.362 |
| Minimum | 288.4 | 322.5 | 299.2 | 329.9 | 350.9 | 324.3 | 304.6 | 325.9 |
| Maximum | 531.1 | 532.1 | 522.4 | 517.8 | 540.7 | 495.0 | 492.7 | 497.6 |
| Range | 242.7 | 209.6 | 223.2 | 187.8 | 189.8 | 170.7 | 188.1 | 171.7 |
| Mean | 416.4 | 429.5 | 397.9 | 418.6 | 421.4 | 423.9 | 395.1 | 418.5 |
| Std. Deviation | 45.0 | 40.1 | 43.0 | 35.2 | 43.5 | 40.8 | 33.0 | 39.1 |
| Variance | 2024.0 | 1608.6 | 1848.1 | 1237.3 | 1895.0 | 1662.9 | 1086.3 | 1527.0 |
| Levene's test | p= 0.965 | | p= 0.896 | | p= 0.625 | | p= 0.285 | |

| **Supplemental Table 2** Childhood trauma type relations with post-trauma stress symptoms and hypothalamus volumes | | | | | | | | |  |
| --- | --- | --- | --- | --- | --- | --- | --- | --- | --- |
|  |  |  |  |  |  |  |  |  |  |
|  | | | Childhood abuse | | | Childhood neglect | | CTQtotal |  |
|  |  |  | CTQea | CTQpa | CTQsa | CTQen | CTQpn |  |  |
| Post-trauma 2-week PCL score ^a^ | | *r* | 0.259 | 0.225 | 0.183 | 0.13 | 0.187 | 0.25 |  |
|  |  | *p* | **0.008*** | **0.024*** | 0.065 | 0.19 | 0.061 | **0.013*** |  |
|  |  | *df* | 101 | 98 | 100 | 101 | 99 | 96 |  |
| Post-trauma 12-months CAPS score ^a^ | | *r* | 0.272 | 0.212 | 0.289 | 0.152 | 0.142 | 0.274 |  |
|  |  | *p* | **0.006*** | **0.034*** | **0.003*** | 0.126 | 0.156 | **0.006*** |  |
|  |  | *df* | 101 | 98 | 100 | 101 | 99 | 96 |  |
| Hypothalamus volume (mm^3^) ^b^ | | | |  |  |  |  |  |  |
| Side | Post-trauma |  |  |  |  |  |  |  |  |
|  | 2 weeks | *r* | -0.187 | -0.236 | -0.056 | -0.304 | -0.157 | -0.24 |  |
| Left |  | *p* | 0.059 | **0.019*** | 0.575 | **0.002*** | 0.118 | **0.018*** |  |
|  |  | *df* | 100 | 97 | 99 | 100 | 98 | 95 |  |
|  | 12 months | *r* | -0.068 | -0.191 | -0.113 | -0.107 | 0.082 | -0.11 |  |
|  |  | *p* | 0.576 | 0.116 | 0.347 | 0.374 | 0.505 | 0.373 |  |
|  |  | *df* | 69 | 67 | 69 | 69 | 67 | 66 |  |
|  | 2 weeks | *r* | -0.203 | -0.281 | -0.175 | -0.287 | -0.223 | -0.272 |  |
| Right |  | *p* | **0.040*** | **0.005*** | 0.079 | **0.003*** | **0.026*** | **0.007*** |  |
|  |  | *df* | 100 | 97 | 99 | 100 | 98 | 95 |  |
|  | 12 months | *r* | -0.181 | -0.35 | -0.258 | -0.138 | -0.11 | -0.264 |  |
|  |  | *p* | 0.132 | **0.003*** | **0.030*** | 0.251 | 0.369 | **0.030*** |  |
|  |  | *df* | 69 | 67 | 69 | 69 | 67 | 66 |  |
| CTQea: emotional abuse, CTQpa: physical abuse, CTQsa: sexual abuse | | | | | | | | |  |
| CTQen: emotional neglect, CTQpn: physical neglect | | | | | | | | |  |
| ^a^: adjusted for age and gender | | | | | | | | |  |
| ^b^: adjusted for age, gender and ICV | | | | | | | | |  |
| *: p< 0.05 as significant level | | | | | | | | |  |
